# Anthocyanin-Rich Nerello Mascalese Pomace Extract: Effects on Osteoblast Differentiation and Bone Matrix Mineralization

**DOI:** 10.3390/foods15173141

**Published:** 2026-09-04

**Authors:** Cristiana Roberta Multisanti, Giovanna Cafeo, Federica Impellitteri, Paola Dugo, Marina Russo, Caterina Faggio, Maria Giovanna Rizzo

**Affiliations:** 1 Department of Veterinary Sciences, University of Messina, Viale Giovanni Palatucci snc, 98168 Messina, Italy; cristiana.multisanti@studenti.unime.it; 2 Institute of Technology c/o Department of Chemical, Biological, Pharmaceutical and Environmental Sciences, University of Messina, Viale G. Palatucci 13, 98168 Messina, Italy; giovanna.cafeo@unime.it (G.C.); paola.dugo@unime.it (P.D.); 3 Department of Chemical, Biological, Pharmaceutical and Environmental Sciences, University of Messina, Viale F. Stagno D’Alcontres 31, 98166 Messina, Italy; fedimpellitteri@unime.it (F.I.); mgrizzo@unime.it (M.G.R.); 4 Chromaleont s.r.l., c/o Department of Chemical, Biological, Pharmaceutical and Environmental Sciences, University of Messina, Viale G. Palatucci 13, 98168 Messina, Italy; 5 Department of Eco-Sustainable Marine Biotechnology, Stazione Zoologica Anton Dohrn, 80122 Naples, Italy

**Keywords:** Nerello Mascalese pomace, phenols, human osteoblasts, bone matrix mineralization

## Abstract

Impaired bone formation and extracellular matrix mineralization contribute to the progressive loss of bone quality associated with aging and skeletal disorders. In this context, the growing interest in sustainable bioactive ingredients for health-related and nutraceutical applications has drawn attention to grape pomace, a phenol-rich byproduct of winemaking. This study investigated the chemical composition and osteogenic potential of Nerello Mascalese pomace extract (NMPE), evaluating its effects on key processes involved in human osteoblast differentiation, extracellular matrix maturation, and mineralized matrix formation. NMPE was chemically characterized by means of liquid chromatographic techniques, revealing a phenolic-rich profile dominated by anthocyanins. The osteogenic effects of NMPE were evaluated by analyzing extracellular matrix mineralization through Alizarin Red S staining, alkaline phosphatase (ALP) enzymatic activity, and the expression of genes involved in osteoblast differentiation and matrix mineralization by quantitative real-time PCR. NMPE promoted osteoblast differentiation and extracellular matrix mineralization, together with coordinated changes in the expression of osteogenesis- and matrix-associated genes. These findings identify Nerello Mascalese pomace as a promising source of bioactive compounds capable of supporting bone-forming processes and strengthen its potential for sustainable valorization in bone-health applications.

## 1. Introduction

Bone is a highly specialized connective tissue that physiologically provides mechanical support, protects organs, serves as the primary reservoir of calcium and phosphate, and contributes to hematopoiesis through its close association with bone marrow [1]. Unlike static tissues, bone undergoes continuous remodeling that continues throughout life. This process is tightly coordinated and preserves skeletal architecture, mechanical integrity, and mineral homeostasis [2]. In this context, bone remodeling is regulated by the dynamic balance between bone resorption by osteoclasts and bone formation by osteoblasts, while osteocytes act as mechano-sensors that coordinate the activity of both cell populations in response to mechanical and biochemical stimuli [3,4]. Clearly, the preservation of bone quality and structural integrity depends on maintaining this cellular balance.

Therefore, bone formation is a regulated process consisting of several stages, including the proliferation of osteoprogenitor cells, differentiation into mature osteoblasts, synthesis of the extracellular matrix, and subsequent mineralization of the matrix [5]. During the process of osteogenic differentiation, osteoblasts express specific molecular markers that reflect their functional maturation. The early stages of differentiation are accompanied by an increase in alkaline phosphatase (ALP) activity, which promotes the generation of an extracellular matrix capable of mineralization through phosphate metabolism [6]. As differentiation progresses, osteoblasts produce structural and regulatory proteins, including bone sialoprotein (IBSP) and osteopontin (SPP1), which contribute to the organization of the extracellular matrix, hydroxyapatite nucleation, and mineral deposition [7]. These events are coordinated by multiple signaling pathways. These include bone morphogenetic proteins (BMPs), such as BMP2, which are involved in the differentiation and maturation of osteoblasts [8,9]. The sequential activation of these mechanisms leads to the formation of mineralized bone tissue.

The disruption of the physiological balance between bone resorption and bone formation shifts skeletal homeostasis toward bone loss, impairing mineralization and increasing susceptibility to fractures [10]. Factors such as aging, menopause, chronic inflammation, oxidative stress, and metabolic disorders can contribute to osteoblast dysfunction [11]. In addition to their role in preserving skeletal integrity, efficient mineralization is also essential for ensuring bone regeneration following fractures, orthopedic surgeries, and bone defects.

Consequently, research is focusing on identifying sustainable strategies capable of preserving osteoblast function, promoting physiological bone formation, and supporting regenerative processes [12,13]. Among these, naturally occurring bioactive compounds are promising candidates due to their biological activity and potential for long-term use. In particular, growing attention is now being paid to the principles of the circular bioeconomy, which promote the sustainable recovery and valorization of agricultural and agro-industrial byproducts as renewable sources of bioactive molecules. In this context, plant-based residues are no longer considered as waste materials, but as sources of phytochemicals with documented biological activity [14,15,16].

Among agro-industrial residues, grape pomace (GP) is the main byproduct of the wine industry and has attracted considerable scientific interest due to its abundance and phytochemical composition. GP consists mainly of skins, seeds, and stem residues, it accounts for approximately 20–30% of the weight of the processed grapes and retains a substantial portion of the fruit’s phenolic compounds after winemaking. These include molecules that have been associated with antioxidant, anti-inflammatory, antimicrobial, cardioprotective, and metabolic health-promoting properties [17,18,19]. In addition to their well-known properties, a growing body of evidence suggests that grape-derived phenols may also have a positive effect on bone metabolism [20]. Among the various classes of phytochemicals found in GP, anthocyanins have attracted particular attention. Indeed, in addition to mitigating oxidative stress and inflammatory responses, anthocyanin-rich plant extracts have been shown to activate osteogenic signaling pathways [21].

However, the osteogenic potential of grape pomace obtained from native cultivars remains largely unexplored. In this context, *Vitis vinifera* L. cv. Nerello Mascalese (NM) is a native Sicilian grapevine widely appreciated for the production of high-quality wines [22]. This specific cultivar produces GP rich in phenolic compounds, as observed in the present study. However, there is insufficient scientific information regarding the biological activity of its pomace in relation to osteoblast function and bone matrix mineralization. Therefore, the present study aimed to integrate the chemical characterization of Nerello Mascalese pomace extract (NMPE) with the evaluation of its osteogenic potential in bone physiology by investigating its effects on osteoblast differentiation, extracellular matrix maturation, and mineralized matrix formation in human hFOB 1.19 cells.

## 2. Materials and Methods

### 2.1. Nerello Mascalese Pomace Extract

Nerello Mascalese pomace extract (NMPE) was obtained from the pomace of *Vitis vinifera* L. cv. Nerello Mascalese provided by a local winery in Sicily, Italy, from a single harvest.

Subsequent to collection, samples were vacuum-packed and stored at −20 °C until further processing. Prior to freeze-drying, the samples were pre-frozen at −80 °C for 24 h. Freeze-drying was performed using an Alpha 2-4 LSCplus freeze-dryer (Martin Christ Gefriertrocknungsanlagen GmbH, Osterode am Harz, Germany) at a condenser temperature of −85 °C and a chamber pressure of 0.05 mbar for 24 h. The resulting freeze-dried material was ground using a laboratory mill and sieved (0.5 mm) to obtain a homogeneous powder.

The extraction of phenolic compounds was carried out according to a previously reported method [23], with minor modifications. Briefly, 500 mg of the powdered sample was extracted with 11 mL of a solution of ethanol/water (50:50, *v*/*v*) with 1% (*v*/*v*) hydrochloric acid. Water and ethanol (HPLC grade, purity ≥ 99.9%), as well as hydrochloric acid (36%) were obtained from Merck Life Science (Merck KGaA, Darmstadt, Germany). Ultrasound-assisted extraction was performed in an ultrasonic bath (Elma Schmidbauer GmbH, Singen, Germany) at 60 kHz and 75 °C for 17 min. The extracts were centrifuged at 4500 rpm for 5 min (Neya XS centrifuge, REMI Sales & Engineering Ltd., Mumbai, Maharashtra, India). The supernatant was collected, filtered through a 0.45 µm membrane filter, and concentrated under a nitrogen stream prior to liquid chromatographic analyses. The extraction procedure was repeated 4 times. The extract yield was 50%, meaning 1.1 g of extract was obtained from 2 g of freeze-dried sample.

### 2.2. Chemical Characterization

Phenolic compounds were analyzed using a Nexera X2 liquid chromatographic system equipped with a photodiode array detector (PDA, SPD-M30A) and a mass spectrometer (LCMS-2020), with an electrospray ionization interface (ESI) operating in both positive and negative ionization mode (Shimadzu, Duisburg, Germany).

Two analytical methods previously developed by Russo and co-workers [24,25] were employed for phenolic acids, flavonoids and anthocyanins.

Analysis of phenolic acids and flavonoids was carried out on a C18 column (150 × 2.1 mm, 2.7 µm) (Merck KGaA, Darmstadt, Germany), using water/formic acid (99.9:0.1, *v*/*v*, solvent A) and water/acetonitrile/formic acid (39.9:60:0.1, *v*/*v*/*v*, solvent B) at a flow rate of 0.2 mL min^−1^. Water and acetonitrile (HPLC-grade, purity ≥ 99.9%), as well as formic acid, were obtained from Merck Life Science (Merck KGaA, Darmstadt, Germany). The gradient elution program was as follows: 0 min, 0% B; 15 min, 20% B; 30 min, 30% B; 50 min, 100% B; 60 min, 100% B.

Anthocyanins were separated on a C18 column (150 × 4.6 mm, 2.7 µm) (Merck KGaA, Darmstadt, Germany), using water/formic acid (90:10, *v*/*v*, solvent A) and water/acetonitrile/formic acid (40:50:10, *v*/*v*/*v*, solvent B) at a flow rate of 1.0 mL min^−1^. The gradient elution program was as follows: 0 min, 12% B; 35 min, 30% B; 36 min, 100% B; 40 min, 100% B. The injection volume was 2 μL for all analyses. PDA detection was carried out over the 200–600 nm range (280, 325, and 518 nm for phenolic acids, flavonoids, and anthocyanins, respectively), with a sampling rate of 1.56 Hz and a time constant of 0.64 s. ESI-MS detection was performed over an m/z range of 100–1000. The operating parameters were set as follows: scan speed, 2000 amu/s; interval, 1.0 s; interface temperature, 250 °C; desolvation line temperature, 250 °C; heat block temperature, 300 °C; nebulizing gas flow rate, 1.5 L min^−1^.

Data acquisition and processing were performed using LabSolutions software (version 5.95, Shimadzu, Duisburg, Germany). Calibration curves of catechin, quercetin, gallic acid, caffeic acid and cyanidin-3-glucoside were used to quantify analytes in the samples based on chromophoric characteristics.

### 2.3. Cell Culture

Human fetal osteoblast cells (hFOB 1.19; American Type Culture Collection, ATCC, Manassas, VA, USA) were used in this study.

hFOB 1.19 cells were selected as the in vitro model because they combine a well-established osteogenic phenotype with good genetic stability and retain their differentiation potential over several passages. These characteristics reduce experimental variability and support greater consistency among osteogenic assays. Compared with primary osteoblasts, which are often affected by donor-dependent differences and a restricted proliferative capacity, hFOB cells represent a more standardized and reproducible platform for investigating the effects of natural bioactive compounds in bone physiology [26,27].

Cells were maintained in a 1:1 mixture of Ham’s F12 medium and Dulbecco’s modified Eagle’s medium (DMEM; D8437, Merck Life Science S.r.l., Milan, Italy), supplemented with 2.5 mM L-glutamine (G7513, Merck Life Science S.r.l.), 10% fetal bovine serum (FBS; F7524, Merck Life Science S.r.l.), and 0.3 mg/mL G418 (4727878001, Merck Life Science S.r.l.) to maintain selective growth. Cells were incubated at 37 °C in a humidified atmosphere containing 5% CO_2_. This temperature was maintained throughout the experimental period, consistent with previous studies [28]. No osteogenic supplements were added to the culture medium; therefore, the effects of NMPE were evaluated under basal culture conditions. The culture medium was replaced twice weekly, and cells were subcultured after reaching approximately 80% confluence.

### 2.4. Cytocompatibility Assay

The cytocompatibility of NMPE in hFOB 1.19 cells was assessed using the 3-[4,5-dimethylthiazol-2-yl]-2,5-diphenyltetrazolium bromide (MTT) reduction assay as an indicator of cellular metabolic activity. Cells were seeded in 96-well microplates (Thermo Fisher Scientific, Seoul, Republic of Korea) at a density of 1 × 10^5^ cells/well. After allowing the cells to adhere, the culture medium was replaced, and cells were incubated with NMPE at final concentrations of 0.97, 1.95, 3.90, 7.80, and 15.62 μg/mL. NMPE was reconstituted from the lyophilized extract as a stock solution of 500 μg/mL and serially diluted to obtain the final treatment concentrations. NMPE stock and working solutions were prepared by dissolving the lyophilized extract directly in cell culture medium (1:1 Ham’s F12/DMEM mixture) at the required concentrations, and sterilized by UV irradiation for 30 min under a laminar flow hood prior to use. Only the concentrations closest to the cytocompatibility threshold are reported, as higher concentrations tested were found to be cytotoxic. Untreated cells were used as the control. After 24 and 48 h of exposure, cells were washed with calcium- and magnesium-free phosphate-buffered saline (PBS). Subsequently, 200 μL of MTT solution, prepared at 1 mg/mL in FBS-free culture medium (M5655, Sigma-Aldrich, St. Louis, MO, USA), was added to each well. After incubation for 2 h at 37 °C and 5% CO_2_, the resulting formazan crystals were solubilized in 200 μL of dimethyl sulfoxide (DMSO) [29]. Absorbance was measured at 540 nm using a microplate reader. Cell viability was calculated relative to untreated cells, which were normalized to 100%, according to the following equation:

$$\mathrm{Cell}\;\mathrm{viability}\;(\%)=\frac{\mathrm{Absorbance}\;\mathrm{of}\;\mathrm{treated}\;\mathrm{cells}}{\mathrm{Absorbance}\;\mathrm{of}\;\mathrm{control}\;\mathrm{cells}}\times 100$$

### 2.5. Extracellular Matrix Mineralization

#### Alizarin Red Staining

The formation of a calcium-rich extracellular matrix was evaluated using Alizarin Red S (ARS; A5533, Merck Life Science S.r.l., Milan, Italy) staining according to the manufacturer’s protocol [30,31]. hFOB 1.19 cells were cultured in the presence of 0.97 μg/mL NMPE for 1 and 14 days. Cells maintained under the same culture conditions without NMPE were used as the control. The culture medium was replaced every 2 days, and the NMPE concentration was maintained constant throughout the experimental period. At each time point, cells were washed with PBS and fixed with 4% paraformaldehyde for 15 min at room temperature. After three washes with distilled water, a 2% Alizarin Red S staining solution was added, and samples were incubated for 30 min at room temperature. The staining solution was then removed, and cells were washed twice with distilled water to eliminate unbound dye. Mineralized calcium deposits were observed using a T Esse Lab inverted microscope V 2.4.

For colorimetric quantification, the bound ARS dye was extracted by adding 300 μL of 10% acetic acid and incubating the samples under shaking for 30 min at room temperature. Cells were scraped and transferred to 1.5 mL tubes, heated at 85 °C for 10 min, and centrifuged at 20,000× *g* for 15 min. Absorbance was measured at 450 nm using a microplate reader AMR-100 (Hangzhou Allsheng Instruments Co.,Ltd., Hangzhou, China).

In parallel to the colorimetric quantification described above, a separate set of cultures was used for time-course image analysis at Days 0, 7, and 14, to visualize the progression of mineralization over time. Quantitative assessment of extracellular matrix mineralization was performed by digital image analysis of Alizarin Red S (ARS)-stained cultures using ImageJ software J 1.54t (National Institutes of Health, Bethesda, MD, USA). Image analysis was conducted at Days 0, 7, and 14 (T0, T7, and T14) in both untreated control (CTRL) and NMPE-treated cultures. Original images were converted to 8-bit grayscale and processed using a standardized analysis workflow. A single threshold value, established from the CTRL images and subsequently applied unchanged to all samples, was used to identify ARS-positive mineralized regions. Pixels above the threshold were automatically segmented and displayed in red to generate binary masks corresponding to mineralized areas. The total number of ARS-positive pixels was then measured and normalized to the total number of pixels in each image (19,961,856 pixels), yielding the percentage of mineralized area (% ARS-positive area). The use of identical acquisition parameters and a fixed threshold ensured objective and reproducible comparisons among treatments and experimental time points.

### 2.6. Alkaline Phosphatase Activity Assay

Alkaline phosphatase enzymatic activity was evaluated in hFOB 1.19 cells cultured in the presence or absence of 0.97 μg/mL NMPE for 1 and 14 days. ALP activity was measured using the Alkaline Phosphatase Assay Kit (ab83369, Abcam, Cambridge, UK), according to the manufacturer’s instructions. The colorimetric assay is based on the enzymatic conversion of *p*-nitrophenyl phosphate into *p*-nitrophenol. Absorbance was measured at 405 nm using a microplate reader. ALP activity was calculated using the standard calibration curve and expressed as U/L.

### 2.7. Osteogenic Gene Expression

The expression of genes involved in osteoblastic differentiation and extracellular matrix mineralization was evaluated by quantitative real-time PCR after 1, 7 and 14 days of treatment with NMPE. Untreated cells were used as the control at each experimental time point. Total RNA was isolated using TRIzol Reagent (Life Technologies, Carlsbad, CA, USA), according to the manufacturer’s instructions, and quantified using a NanoDrop ND-1000 UV spectrophotometer (White Bear Photonics, LLC, White Bear Lake, MN, USA). The reverse transcription (RT) was carried out in a 20 mL reaction mixture, containing 1× reaction buffer, 0.5 mM dNTP, 20 pmol primers, 3 mM MgCl2, 20 U RNAase inhibitor, and 200 U Improm II reverse transcriptase (Promega Corporation, Madison, WI, USA). Amplification was carried out using a 7500 Fast Real-Time PCR System under the following conditions: initial denaturation at 95 °C for 3 min, followed by 35 cycles of denaturation at 95 °C for 15 s, annealing at 55 °C for 30 s, and extension at 72 °C for 15 s. A melting curve analysis was performed using the instrument’s default settings.

Relative gene expression was calculated using the $2^{-\Delta \Delta Ct}$ method. *GAPDH* was used as the reference gene, and results were expressed as fold changes relative to the corresponding untreated control, normalized to 1 [32,33]. Primer specificity and efficiency were verified prior to analysis. Oligonucleotide sequences are reported in Table 1.

### 2.8. Statistical Analysis

The data were derived from three independent experiments conducted in triplicate. All the data are expressed as the means ± standard deviation. Data were analyzed using one or two-way ANOVA followed by Bonferroni’s correction unless otherwise stated. These analyses were performed using GraphPad Prism 8. A *p* value of 0.05 or less was regarded as significant.

## 3. Results

### 3.1. Chemical Characterization

The phytochemical characterization of the Nerello Mascalese grape pomace extract by HPLC-PDA-MS revealed the presence of eight anthocyanins, six phenolic acids and seven flavonoids. The detailed phenolic composition is reported in Table 1. Anthocyanins represented the predominant class (206.7 ± 12.3 mg kg^−1^), according to literature data [34,35,36]. Among these, malvidin-3-glucoside was the most abundant compound (138.9 ± 11.2 mg kg^−1^), followed by peonidin-3-glucoside (23.7 ± 0.1 mg kg^−1^) and petunidin-3-galactoside (18.2 ± 1.0 mg kg^−1^), while acetylated derivatives were present at lower levels (3–5 mg kg^−1^). Phenolic acids were the second most represented group, with gallic acid as the major component (20.7 ± 0.2 mg kg^−1^). Flavonoids were detected at lower concentrations, totaling 11.5 ± 0.2 mg kg^−1^, with isorhamnetin-3-glucoside (3.4 ± 0.1 mg kg^−1^) and rutin (3.7 ± 0.1 mg kg^−1^) as the main constituents. Overall, the total phenolic content reached 304.5 ± 13.6 mg kg^−1^, confirming that Nerello Mascalese pomace is a rich source of bioactive compounds, particularly anthocyanins.

### 3.2. Cytocompatibility

**Figure 1:**
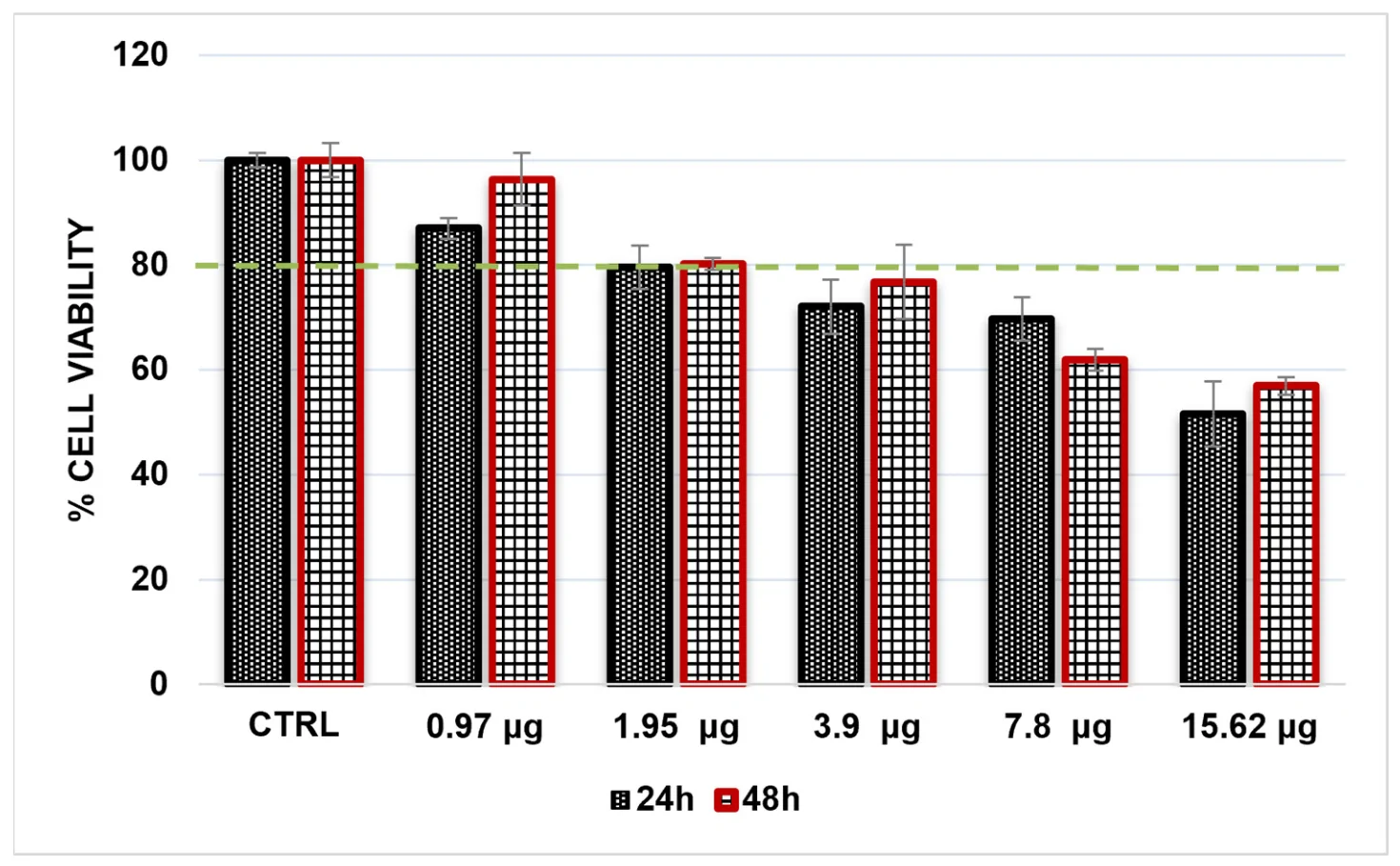
Effect of NMPE on hFOB 1.19 cell viability. Cell viability was evaluated by MTT assay after treatment with increasing concentrations of Nerello Mascalese pomace extract for 24 and 48 h. Untreated cells were used as the control and normalized to 100%. Results are expressed as mean ± SD. The green dashed line indicates the 70–80% cell viability threshold, in line with ISO 10993-5 guidelines.

The cytocompatibility of Nerello Mascalese pomace extract (NMPE) was evaluated in hFOB 1.19 cells using the MTT assay after 24 and 48 h of exposure to concentrations ranging from 0.97 to 15.62 μg/mL (Figure 1). Specifically, as reported in Table 2, NMPE cytocompatibility was evaluated by subjecting cells to absolute amounts of phenolic compounds ranging from 0.034 to 0.53 µg. Untreated cells were used as the control and their viability was normalized to 100%.

After 24 h, NMPE induced a concentration-dependent reduction in cell viability, with values of 87.01 ± 2.03%, 79.62 ± 4.11%, 72.05 ± 4.10%, 69.75 ± 4.10%, and 51.62 ± 6.22% at 0.97, 1.95, 3.90, 7.80, and 15.62 μg/mL, respectively. A similar concentration-dependent trend was observed after 48 h. Cell viability was 96.30 ± 5.03% at 0.97 μg/mL and approximately 80% at 1.95 μg/mL, whereas higher concentrations progressively reduced cell viability to 76.78 ± 7.13%, 61.91 ± 2.10%, and 56.95 ± 1.67% at 3.90, 7.80, and 15.62 μg/mL, respectively.

Among the tested concentrations, 0.97 μg/mL showed the most favorable cytocompatibility profile. At this concentration, cell viability increased from 87.01% at 24 h to 96.30% at 48 h, approaching the value observed in untreated cells. Based on the preservation of cell metabolic activity and the higher viability observed after prolonged exposure, 0.97 μg/mL NMPE was selected as the working concentration for the subsequent osteogenic experiments.

### 3.3. Extracellular Matrix Mineralization

**Figure 2:**
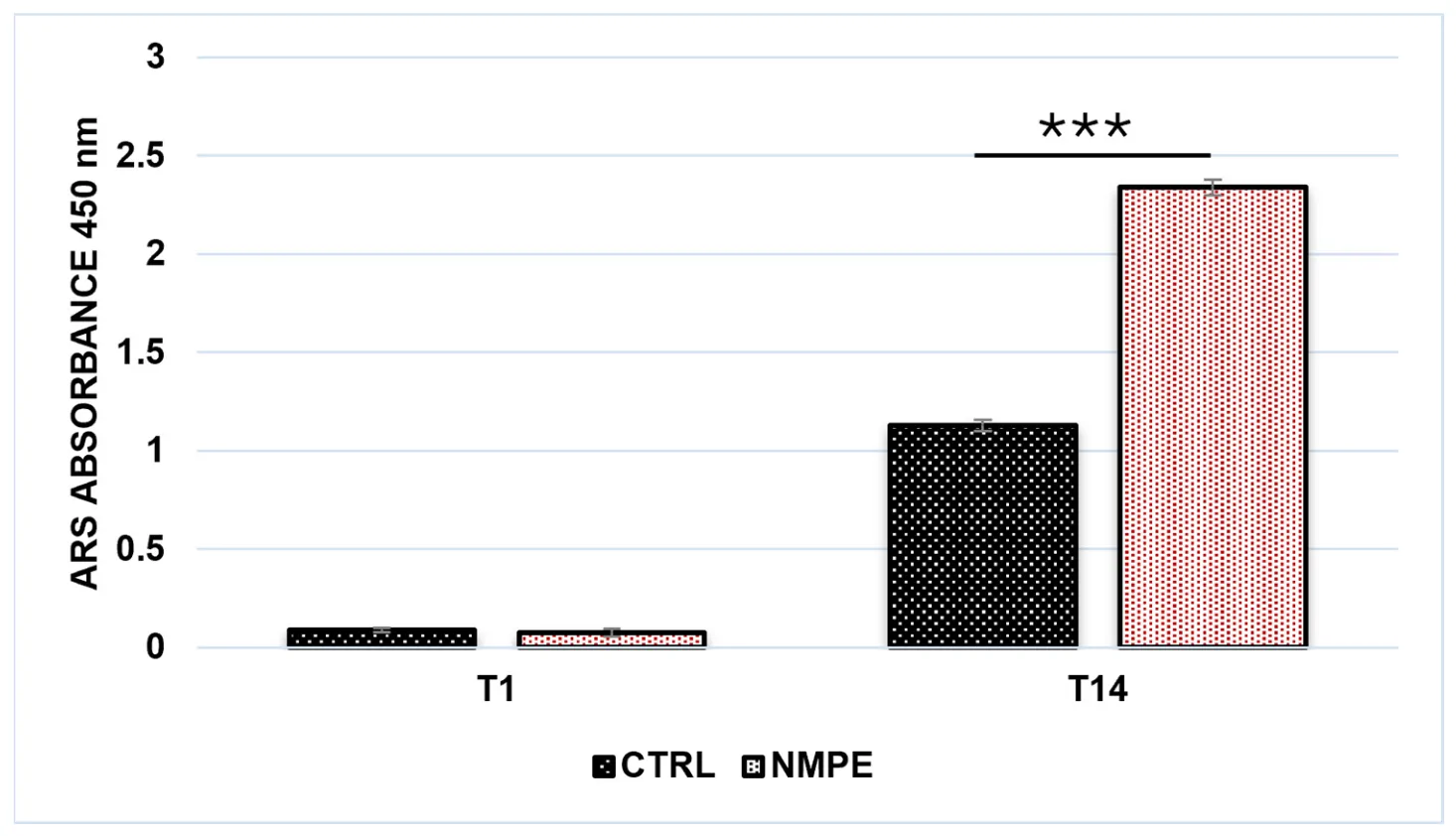
Effect of NMPE on extracellular matrix mineralization. Alizarin Red S staining was used to evaluate calcium deposition in untreated cells (CTRL) and cells treated with Nerello Mascalese pomace extract (NMPE) at Day 1 (T1) and Day 14 (T14). The bound dye was quantified by measuring absorbance at 450 nm. Data are presented as mean ± standard deviation (SD). Statistical analysis was performed using one-way ANOVA followed by Bonferroni’s multiple-comparison test. Asterisks indicate statistically significant differences compared with the untreated control (*** *p* < 0.001).

Extracellular matrix mineralization was evaluated by Alizarin Red S (ARS) staining in hFOB 1.19 cells cultured in the presence or absence of NMPE for 1 and 14 days (Figure 2).

At T1, low ARS absorbance values were detected in both experimental groups, with values of 0.090 ± 0.011 in control cells and 0.077 ± 0.020 in NMPE-treated cells, indicating limited calcium deposition at the early time point. After 14 days, ARS absorbance markedly increased in both groups, reaching 1.13 ± 0.03 in the control and 2.34 ± 0.04 in NMPE-treated cells.

Notably, at T14, the ARS signal detected in NMPE-treated cells was approximately two-fold higher than that observed in untreated cells. These results indicate that prolonged exposure to NMPE promotes calcium-rich extracellular matrix deposition and enhances the mineralization process in hFOB cells.

**Figure 3:**
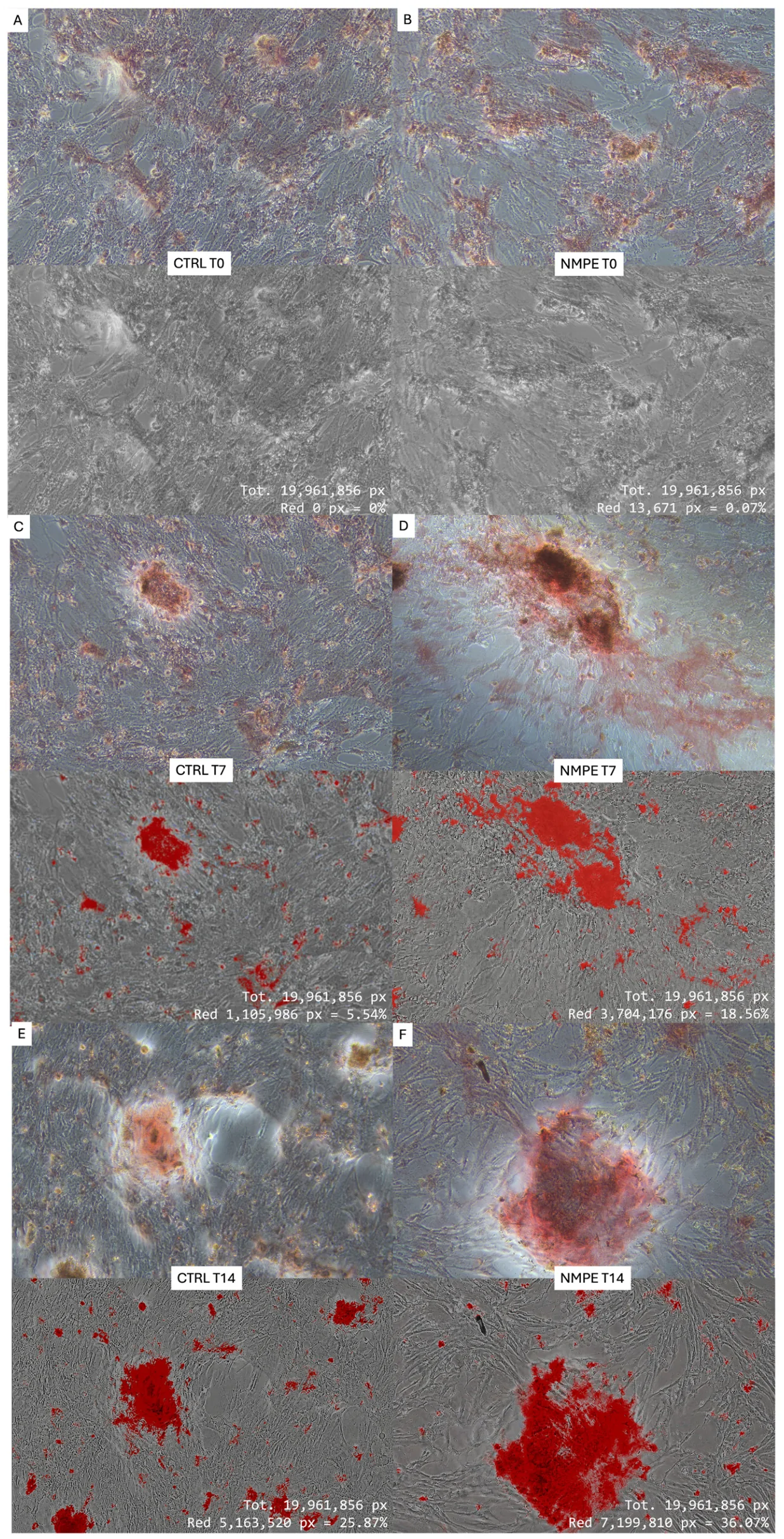
Representative images of extracellular matrix mineralization and ImageJ-based visualization of Alizarin Red S (ARS)-positive areas in hFOB 1.19 cells. Representative ARS-stained micrographs of untreated cells (CTRL) and cells exposed to Nerello Mascalese Pomace Extract (NMPE) at T0 (**A**,**B**), T7 (**C**,**D**), and T14 (**E**,**F**). For each representative field, the corresponding ImageJ-processed image is shown below, with ARS-positive mineralized areas highlighted in red. Images are representative of three microscopic fields from each of three independent experiments (*n* = 3 biological replicates, three fields per replicate). Figure 3 is intended for qualitative visualization of ARS-positive areas, whereas quantitative assessment of matrix mineralization is reported in Figure 2.

To complement the colorimetric assessment of mineralization, ARS-stained micrographs were subjected to quantitative image analysis using a standardized color threshold. Images were acquired under identical magnification and resolution settings, allowing the proportion of ARS-positive pixels to be compared across experimental conditions (Figure 3). At T0, mineralized areas were negligible in both control and NMPE groups. At T7, the percentage of red pixels increased to approximately 5–6% in control cultures and 18–19% in NMPE-treated cells. A further increase was observed at T14, reaching approximately 25–26% in untreated cells and 36% following NMPE treatment. Finally, the image analysis revealed a progressive accumulation of calcium-rich deposits over time, with a greater ARS-positive area in NMPE-treated cultures from T7 onward. These results confirmed the colorimetric ARS measurements and provide additional evidence that NMPE enhances mineralized extracellular matrix formation in hFOB 1.19 cells.

### 3.4. Alkaline Phosphatase Enzymatic Activity

**Figure 4:**
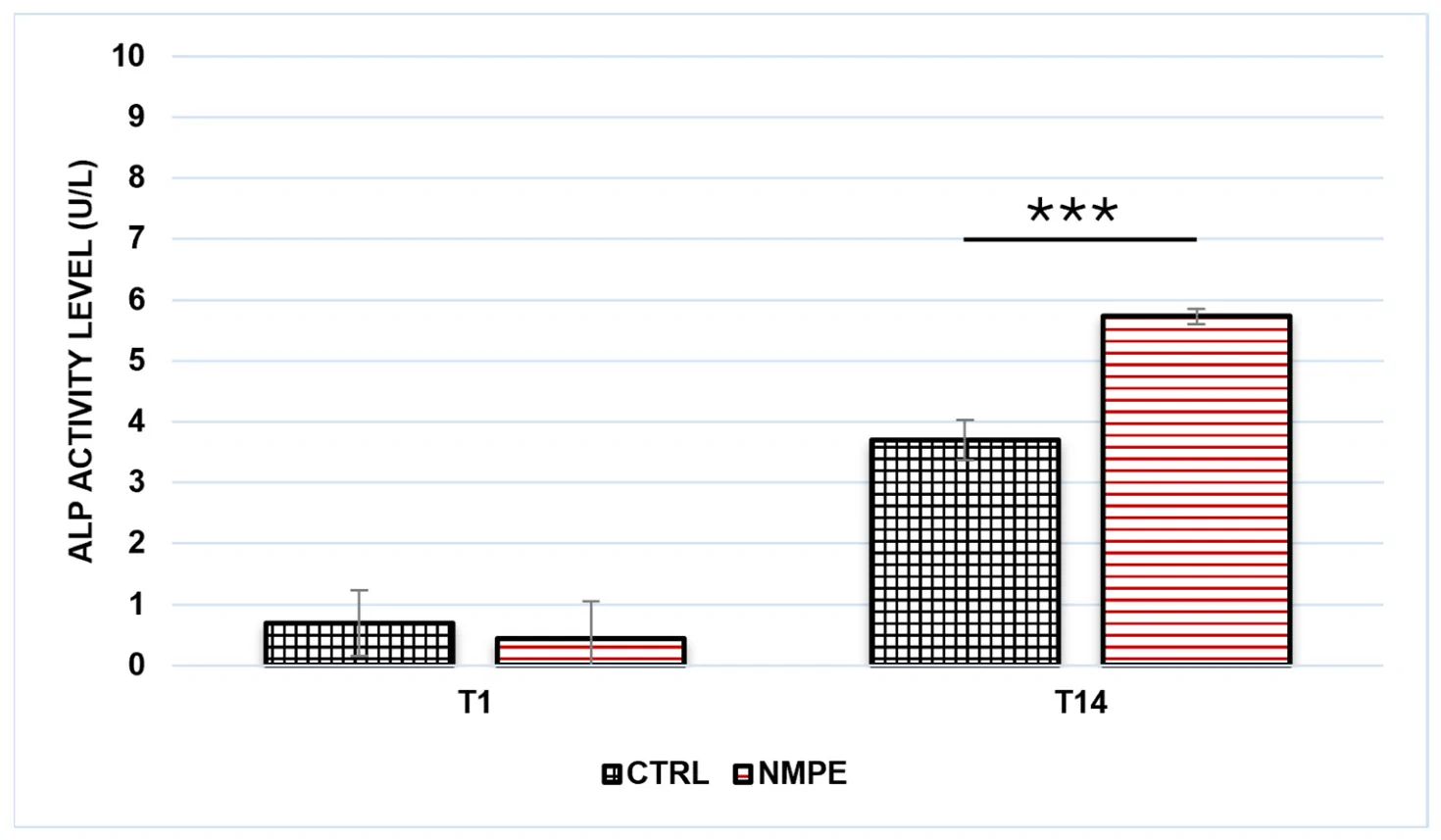
Effect of NMPE on alkaline phosphatase enzymatic activity in hFOB 1.19 cells. Alkaline phosphatase (ALP) activity was measured in untreated cells (CTRL) and cells treated with Nerello Mascalese pomace extract (NMPE) at Day 1 (T1) and Day 14 (T14). ALP activity is expressed as U/L. Data are presented as mean ± standard deviation (SD). Statistical analysis was performed using one-way ANOVA followed by Bonferroni’s multiple-comparison test. Asterisks indicate statistically significant differences compared with the untreated control (*** *p* < 0.001).

Alkaline phosphatase (ALP) enzymatic activity was evaluated in hFOB 1.19 cells cultured in the presence or absence of NMPE for 1 and 14 days (Figure 4). At T1, ALP activity remained low in both experimental groups, with values of 0.70 ± 0.54 U/L in control cells and 0.44 ± 0.61 U/L in NMPE-treated cells. After 14 days, a marked time-dependent increase in ALP activity was observed in both groups. In particular, ALP activity reached 3.70 ± 0.33 U/L in control cells and 5.73 ± 0.13 U/L in cells treated with NMPE.

At T14, NMPE-treated cells exhibited approximately 1.5-fold higher ALP activity than untreated cells. These results indicate that prolonged exposure to NMPE enhances ALP enzymatic activity in hFOB cells, consistent with the increased extracellular matrix mineralization observed by ARS staining.

### 3.5. Osteogenic Gene Expression

**Figure 5:**
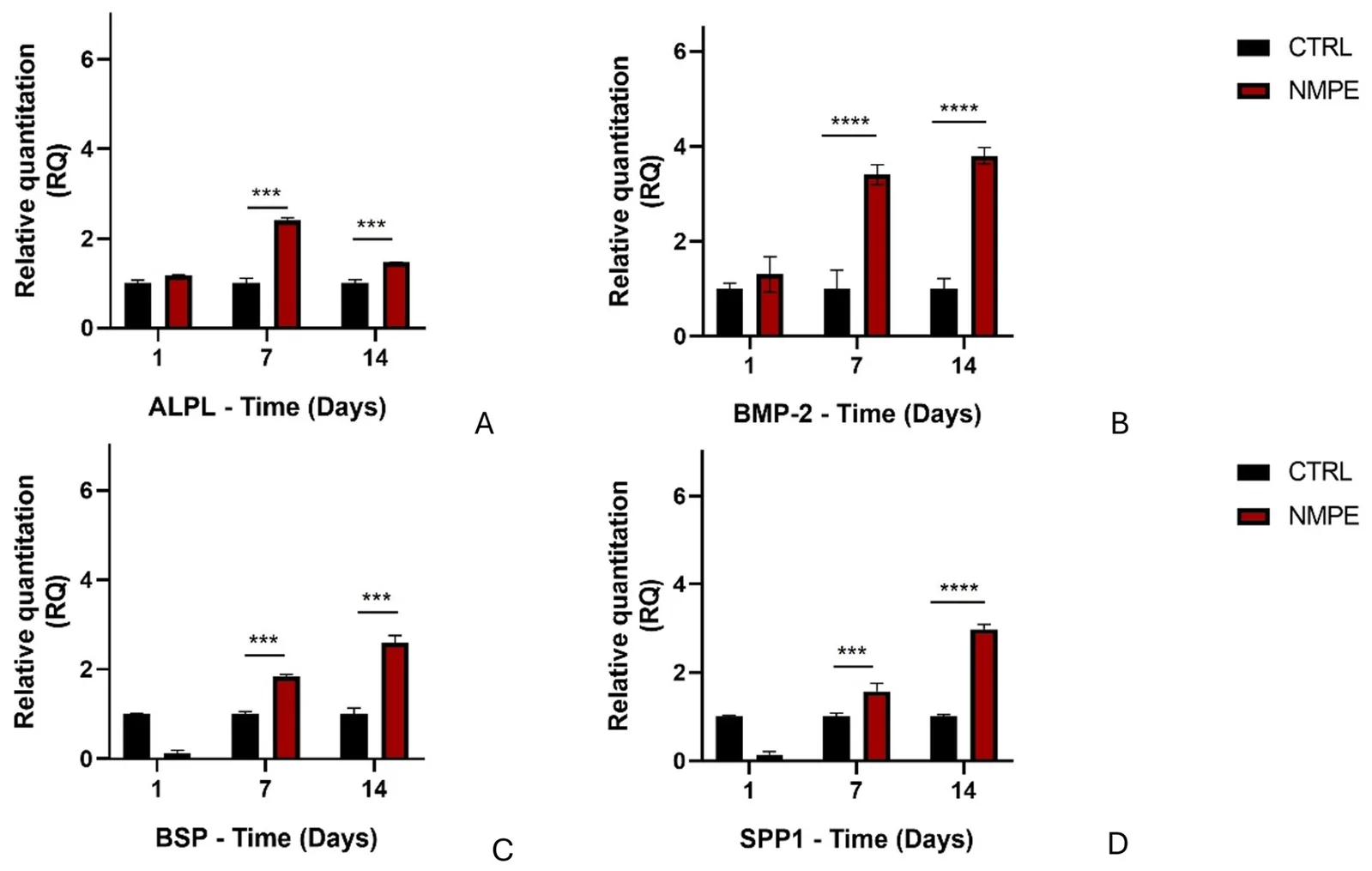
Effect of NMPE on osteogenic gene expression. Relative expression levels of (**A**) alkaline phosphatase, biomineralization-associated (ALPL), (**B**) bone morphogenetic protein 2 (BMP2), (**C**) integrin-binding sialoprotein (IBSP; bone sialoprotein), and (**D**) secreted phosphoprotein 1 (SPP1; osteopontin) were evaluated after 1, 7, and 14 days in untreated cells (CTRL) and cells treated with Nerello Mascalese pomace extract (NMPE). The statistical analysis was reported as *** *p* < 0.001, **** *p* < 0.0001. The data were derived from three independent experiments conducted in triplicate.

The effect of Nerello Mascalese pomace extract (NMPE) on osteogenic differentiation was investigated by evaluating the expression of *ALPL*, *BMP2*, *IBSP*, and *SPP1* in hFOB 1.19 cells after 1, 7, and 14 days of treatment with NMPE (Figure 5). These genes were selected to represent complementary phases and functions of the osteogenic process. *ALPL* is associated with osteoblast differentiation and the generation of a mineralization-competent extracellular matrix, whereas *BMP2* acts as a major osteoinductive signal promoting osteoblast differentiation and bone formation. *IBSP*, encoding bone sialoprotein, contributes to cell adhesion and hydroxyapatite nucleation, while *SPP1*, encoding osteopontin, is involved in cell–matrix interactions and in the organization and remodeling of the mineralized extracellular matrix. Gene expression was reported as relative quantification (RQ), with the untreated control normalized to 1 at each time point.

NMPE treatment induced a time-dependent modulation of *ALPL* expression. At Day 1, a modest increase was observed in NMPE-treated cells, with an RQ value of 1.176 ± 0.037 compared with the control. Expression increased markedly at Day 7, reaching its maximum value of 2.400 ± 0.161. At Day 14, *ALPL* expression decreased to 1.470 ± 0.006, although it remained higher than the corresponding control. This temporal profile indicates that the effect of NMPE on *ALPL* was most pronounced during the intermediate phase of osteogenic differentiation. The peak observed at Day 7, followed by a reduction at Day 14, is consistent with the role of ALPL as an early-to-intermediate marker involved in preparing the extracellular matrix for subsequent mineral deposition.

A progressive and sustained increase in *BMP2* expression was observed following NMPE treatment. At Day 1, *BMP2* expression was 1.30 ± 0.40-fold relative to the control. A marked upregulation was detected at Day 7, when the RQ value increased to 3.40 ± 0.20, and was further maintained at Day 14, reaching 3.80 ± 0.20. Unlike the transient response observed for *ALPL*, *BMP2* expression progressively increased throughout the experimental period.

This pattern suggests that NMPE supports a prolonged increase in the expression of osteoinductive genes associated with osteoblast differentiation and the progression toward a mineralizing phenotype.

The expression of *IBSP*, encoding bone sialoprotein, showed a delayed time-dependent response to NMPE treatment. At Day 1, *IBSP* expression was markedly lower in NMPE-treated cells than in the control, with an RQ value of 0.124 ± 0.172. However, expression increased above control levels at Day 7, reaching 1.841 ± 0.099, and further increased at Day 14 to 2.587 ± 0.392.

The absence of an early increase, followed by progressive upregulation at the intermediate and late time points, indicates that NMPE primarily modulates *IBSP* during the phases associated with extracellular matrix maturation and mineral deposition. Bone sialoprotein is an important component of the bone matrix and contributes to the nucleation of hydroxyapatite crystals.

A similar delayed response was observed for *SPP1*. At Day 1, NMPE-treated cells showed a low expression value of 0.130 ± 0.199 relative to the control. Expression subsequently increased to 1.575 ± 0.432 at Day 7 and reached its maximum value of 2.970 ± 0.273 at Day 14.

The progressive increase in *SPP1* expression after prolonged NMPE exposure suggests a greater involvement of this marker during the later stages of osteogenic differentiation. SPP1, which encodes osteopontin, is involved in cell–matrix interactions and in the organization and remodeling of the extracellular matrix during mineralization.

Finally, NMPE induced a coordinated temporal modulation of osteogenic markers. *ALPL* expression peaked at Day 7 and subsequently decreased while remaining above control levels, whereas *BMP2* showed a sustained increase from Day 7 to Day 14. In contrast, the bone matrix-associated genes *IBSP* and *SPP1* were initially downregulated at Day 1 and progressively increased at the intermediate and late time points.

These results suggest a differential treatment response across the examined time points characterized by sustained upregulation of BMP2, intermediate induction of ALPL, and subsequent upregulation of genes involved in extracellular matrix organization, maturation, and mineral deposition. This transcriptional profile is consistent with the increased ALP enzymatic activity and ARS-detectable calcium deposition observed after 14 days of NMPE treatment.

## 4. Discussion

The osteogenic effects observed following NMPE exposure may be related to the combined activity of the phenolic constituents identified in the extract [37]. The chemical profile was dominated by anthocyanins, particularly malvidin-3-glucoside, with smaller contributions from phenolic acids and flavonoids. Anthocyanins as a class have been linked to modulation of osteoblast differentiation and bone-related signaling pathways, including ERK1/2-mediated mechanisms demonstrated for cyanidin-3-glucoside; whether malvidin-3-glucoside, the dominant anthocyanin in NMPE, acts through a comparable pathway has not yet been established and warrants direct investigation [37,38].

The enhanced mineralized matrix formation observed in NMPE-treated cultures suggests that the phenolic-rich extract can increase the mineralization competence of osteoblasts under basal culture conditions. This effect may reflect the combined modulation of osteoblast differentiation and extracellular matrix maturation, processes that are regulated by multiple phenolic-sensitive signaling mechanisms [38,39,40].

Notably, NMPE induced osteogenic responses at a relatively low nominal extract concentration (0.97 μg/mL) under basal culture conditions. Previous studies investigating grape pomace polyphenolic extracts reported osteogenic effects at 10–20 μg/mL in human mesenchymal stem cells [20], while a recent study identified 5 μg GAE/mL as the lowest effective concentration capable of enhancing matrix mineralization [40]. Although direct quantitative comparisons are limited by differences in extract standardization, cellular models, and culture conditions, these findings suggest that NMPE retains osteogenic activity at a low concentration and in the absence of conventional osteogenic supplements.

This unusually low effective concentration may reflect the high relative purity of the anthocyanin fraction in NMPE, differences in extraction methodology compared to previous studies, and/or greater sensitivity of the hFOB 1.19 model.

ALP favors hydroxyapatite formation by reducing extracellular pyrophosphate, a mineralization inhibitor, and increasing the availability of inorganic phosphate [41]. Its increase in parallel with ARS staining therefore provides a functional link between osteoblast differentiation and calcium-rich matrix deposition. Interestingly, the largest treatment-associated increase in *ALPL* expression was observed at Day 7, whereas the greatest difference in ALP enzymatic activity was detected at Day 14. Although these measurements were obtained at different experimental time points, this pattern is compatible with the possibility that transcriptional modulation precedes changes detectable at the enzymatic level. Future studies will incorporate normalization of ARS and ALP measurements to cell number, protein, or DNA content to strengthen data interpretation.

The increased *BMP2* expression observed at the later examined time points provides a possible molecular basis for the osteogenic effects of NMPE. *BMP2* is a key regulator of osteoblast differentiation, ALP expression, and bone matrix protein production, *BMP2* can regulate osteoblast differentiation through pathways including BMP/Smad and WNT-dependent signaling. Its temporal pattern offers a plausible upstream framework for the transient induction of *ALPL* and the later increase in matrix-associated genes.

The increased expression of *IBSP* and *SPP1* at the later examined time points may indicate that NMPE also influences extracellular matrix organization and maturation [42]. The parallel induction of *IBSP* and *SPP1*, together with higher ALP activity and ARS staining, supports the formation and organization of a more mature mineralized extracellular matrix [43].

These findings support the interpretation that NMPE influences several interconnected components of osteoblast function. The modulation of BMP2 expression may indicate an effect on osteogenic signaling, while changes in ALPL expression and ALP activity are consistent with increased mineralization competence. Lastly, NMPE can be considered an anthocyanin-rich phytocomplex with osteogenic potential. Future studies will investigate the effects of NMPE in vivo, including a recognized osteogenic positive control. This study relied on gene expression profiling without protein-level or pathway-specific validation (e.g., Western blot, pathway inhibitors, RUNX2/NRF2/ER-β assays).

Beyond the BMP/Smad and WNT pathways discussed above, malvidin itself has been shown to modulate osteogenic differentiation of human mesenchymal stem cells [44]. Anthocyanins as a class have also been linked to Nrf2-ARE antioxidant signaling and phytoestrogen-like activity via estrogen receptor-β (ER-β) in osteoblasts. Further characterization of the intracellular redox state and upstream osteogenic signaling pathways will be important to clarify the molecular mechanisms underlying the observed effects. Batch-to-batch phenolic variability was not assessed and should be addressed before translational applications are considered.

## 5. Conclusions

Nerello Mascalese pomace extract promoted a coordinated osteogenic response in hFOB 1.19 cells, combining preserved metabolic activity at the selected concentration with increased alkaline phosphatase activity, calcium deposition, and time-dependent modulation of genes involved in osteoblast differentiation and matrix maturation. These convergent responses suggest that NMPE supports the physiological sequence of bone formation, from osteoblast differentiation to extracellular matrix maturation and mineral deposition. From a food chemistry perspective, these findings add biological value to Nerello Mascalese pomace, transforming a winemaking byproduct into a potential source of bone-active compounds. Although validation in more complex experimental models is required, NMPE promoted osteogenic marker expression and matrix mineralization in hFOB 1.19 cells in vitro, supporting its potential as a source of bioactive compounds for further investigation in bone-health applications.

## Figures and Tables

**Table 1:** Primer sequences (5′–3′) used for qRT-PCR analysis.

Protein Name	Target Gene	Protein Function	Forward	Reverse
Glyceraldehyde 3-phosphate dehydrogenase	GAPDH	Housekeeping genes	AACAGCGACACCCACTCCTC	CATACCAGGAAATGAGCTTGACAA
Alkaline phosphatase	ALPL	Bone mineralization	ACCATTCCCACGTCTTCACATTT	AGACATTCTCTCGTTCACCGCC
Bone Sialoprotein	IBSP	Cell adhesion	GGCAGTAGTGACTCATCCGAAG	GAAAGTGTGGTATTCTCAGCCTC
Osteopontin	SPP1	Bone matrix remodeling	GCAGACCTGACATCCAGTACC	GATGGCCTTGTATGCACCATTC
Bone Morphogenetic protein 2	BMP2	Osteoblast differentiation and bone formation	ACTCGAAATTCCCCGTGACC	CCACTTCCACCACGAATCCA

**Table 2:** Concentration (mg kg^−1^ ± standard deviation) of phenolic compounds in freeze-dried Nerello mascalese grape pomace.

Phenolic Compounds	mg kg^−1^
**Anthocyanins**	
Delphinidin-3-glucoside ^a^	7.87 ± 0.15
Cyanidin-3-glucoside	6.50 ± 0.41
Petunidin-3-galactoside ^a^	18.16 ± 1.02
Peonidin-3-glucoside ^a^	23.71 ± 0.07
Malvidin-3-glucoside	138.87 ± 11.20
Delphinidin-3-(6″-acetyl)-glucoside ^a^	3.03 ± 0.24
Cyanidin-3-(6″-acetyl)-glucoside ^a^	3.43 ± 0.16
Petunidin-3-(6″-acetyl)-glucoside ^a^	5.12 ± 0.04
*Tot. Anthocyanins*	*206.70 ± 12.33*
**Phenolic acids**	
Syringic acid ^b^	15.35 ± 0.03
Ferulic acid ^b^	14.86 ± 0.02
Sinapic acid ^b^	15.80 ± 0.01
Citric acid ^b^	15.19 ± 0.01
Gallic acid	20.70 ± 0.15
Caffeoyl tartaric acid ^c^	4.39 ± 0.03
*Tot. Phenolic acids*	*86.29 ± 0.14*
**Flavonoids**	
Kaempferol ^d^	1.10 ± 0.03
Catechin	0.60 ± 0.01
Epicatechin ^e^	1.23 ± 0.03
Quercetin	1.14 ± 0.00
Isoquercetin ^d^	0.34 ± 0.01
Isorhamnetin-3-glucoside ^d^	3.36 ± 0.05
Rutin ^d^	3.72 ± 0.06
*Tot. Flavonoids*	*11.49 ± 0.18*
***Tot. Phenolic compounds***	***304.47 ± 13.59***

Phenolic compounds were quantified based on calibration curves of the correspondent standard compounds: ^a^ cyanidin-3-glucoside, ^b^ gallic acid, ^c^ caffeic acid, ^d^ quercetin; ^e^ catechin.

## Data Availability

All data supporting the findings of this study are available within the document.

## Abbreviations

The following abbreviations are used in this manuscript:

ALP	Alkaline Phosphatase
ALPL	Alkaline Phosphatase, Biomineralization Associated
ARS	Alizarin Red S
BMP2	Bone Morphogenetic Protein 2
DMEM	Dulbecco’s Modified Eagle Medium
FBS	Fetal Bovine Serum
GAPDH	Glyceraldehyde-3-Phosphate Dehydrogenase
GP	Grape Pomace
HPLC-PDA-MS	High-Performance Liquid Chromatography–Photodiode Array–Mass Spectrometry
IBSP	Integrin-Binding Sialoprotein
MTT	3-(4,5-Dimethylthiazol-2-yl)-2,5-Diphenyltetrazolium Bromide
NM	Nerello Mascalese
NMPE	Nerello Mascalese Pomace Extract
PBS	Phosphate-Buffered Saline
qRT-PCR	Quantitative Real-Time Polymerase Chain Reaction
SPP1	Secreted Phosphoprotein 1
